# Supplementary material for: Molecular Polymorphism of tau aggregates in Pick’s disease
Source: bioRxiv. 2024 Nov 19:2024.11.19.624253. Preprint. [Version 1] doi: 10.1101/2024.11.19.624253 (PMC11601565; doi:10.1101/2024.11.19.624253)
Supplement: Supplement 1 [file NIHPP2024.11.19.624253v1-supplement-1.pdf]

bioRxiv preprint doi: https://doi.org/10.1101/2024.11.19.624253; this version posted November 19, 2024. The copyright holder for this preprint (which was not certified by peer review) is the author/funder, who has granted bioRxiv a license to display the preprint in perpetuity. It is made available under aCC-BY 4.0 International license.

Appendix: Molecular Polymorphism of tau aggregates in Pick's disease

Jiliang Liu[a*], Theresa Connors Stewart[b], Bradley T. Hyman[b], Manfred Burghammer[a], Marine Cotte[a], Lee Makowski[c*].

d. European Synchrotron Radiation Facility, Grenoble 38043, France;
e. Department of Neurology, Massachusetts General Hospital, Charlestown, MA 02129, USA;
f. Bioengineering Department, Northeastern University, Boston, MA 02115, USA.

The nanoscale packing of tau filaments determined by SAXS.

The SAXS regime contains information on structural features of the scattering particles that have characteristic dimensions in the 1-100 nm scale. At this level, SAXS can be considered as scattering from a continuous distribution of electron density and approximated by a simplified model. Based on the structure of cross-β tau filament of Pick's disease as determined by cryoEM (1), we constructed a parallelepiped model as shown in **Figure S4a**, with a cross section of 10 x 100 Å for the 'narrow pick filament' (NPF) and 10 x 200 Å for the 'wide pick filament' (WPF). The form factor of a parallelepiped can be calculated as:

$$F(q) = \frac{sin\left(q\frac{A}{2}\right)}{q\frac{A}{2}}\frac{sin\left(q\frac{B}{2}\right)}{q\frac{B}{2}}\frac{sin\left(q\frac{C}{2}\right)}{q\frac{C}{2}}$$

Considering the centroid symmetry of the parallelepiped, the 1D intensity profile can be calculated as the spherically averaged squared form factor as:

$$I(q) = \langle F(q)^2 \rangle$$

$$= scale * \int_0^{\pi}\int_0^{2\pi}\left[\frac{sin\left(q\frac{A}{2}sin\phi sin\varphi\right)}{q\frac{A}{2}sin\phi sin\varphi}\frac{sin\left(q\frac{B}{2}cos\phi sin\varphi\right)}{q\frac{B}{2}cos\phi sin\varphi}\frac{sin\left(q\frac{C}{2}cos\varphi\right)}{q\frac{C}{2}cos\varphi}\right]^2 sin(\varphi)d\phi d\varphi$$

…………………………..1

The slope of the SAXS intensity provides an approximation for the dimensions of these filaments. **Figure S4 a)** and **b)** show a parallelepiped model with transverse dimension of 10 x 100 Å (A, B

bioRxiv preprint doi: https://doi.org/10.1101/2024.11.19.624253; this version posted November 19, 2024. The copyright holder for this preprint (which was not certified by peer review) is the author/funder, who has granted bioRxiv a license to display the preprint in perpetuity. It is made available under aCC-BY 4.0 International license.

direction in **Figure S4a**) and 150 Å in longitudinal direction (C direction in **Figure S4a**) that gives rise to a profile with a slope comparable to the SAXS pattern from fibrillar tau (**Figure S4c**). This structural model is consistent with the NPF model of tau filament for Pick's disease (Falcon et al. 2018). However, the features of the SAXS intensity pattern at 0.07 Å^{-1} and 0.2 Å^{-1} (**Figure 3**) indicate that, *in situ*, tau filaments further aggregate to form larger scale hierarchical structures within the lesion. The interference scattering arising from this higher order structure can be calculated as:

$$I(q) = \langle \left[F(q) \left(\sum_{i=0}^{N} \sum_{j=0}^{M} S_{ij}(q) \right) \right]^2 \rangle$$

where $S_{ij}(q) = \int \delta(\vec{x}_{ij}) e^{\vec{q}\vec{x}} d\vec{x} = e^{\vec{q}\vec{x}_{ij}}$ is the Fourier transform of the translation vector in real space. Thus, the spherically averaged intensity profile for the model of higher order aggregation of tau, shown in the Figure **S4c**, is calculated as:

$$I(q) = scale * \int_0^{\pi} \int_0^{2\pi} \left[\frac{sin\left(q\frac{A}{2} sin\phi sin\varphi\right)}{q\frac{A}{2} sin\phi sin\varphi} \frac{sin\left(q\frac{B}{2} cos\phi sin\varphi\right)}{q\frac{B}{2} cos\phi sin\varphi} \frac{sin\left(q\frac{C}{2} cos\varphi\right)}{q\frac{C}{2} cos\varphi} \left(1 + e^{\vec{q}\vec{x}_1} + e^{\vec{q}\vec{x}_2} + e^{i\vec{q}\vec{x}_3}\right) \right]^2 sin(\varphi) d\phi d\varphi$$

………………………..2

where x1= [30, 0, 0], x2 = [80, 0, 0,] and x3= [110, 0, 0]. **Figure S4d** shows that a SAXS profile calculated from a model including four parallelepiped fibrils with 10 x100 Å transverse structure and 20Å in the axial direction exhibits interference scattering consistent with experimental SAXS in both maximum position and log-log slope. However, the predicted sharpness of the calculated intensity is far greater than experimental data, suggesting polymorphism in the higher-order structural organization. The polymorphism in the the higher-order organization of tau fibrils was incorporated into the model as:

$$I(q) = \iint p(d1, d2) I_{d1d2} dp$$

$$p = \frac{1}{N_{d_1} N_{d_2}}$$

bioRxiv preprint doi: https://doi.org/10.1101/2024.11.19.624253; this version posted November 19, 2024. The copyright holder for this preprint (which was not certified by peer review) is the author/funder, who has granted bioRxiv a license to display the preprint in perpetuity. It is made available under aCC-BY 4.0 International license.

where d1 was given values of [20Å, 25Å, 30Å, 35Å, 40Å] and d2 [85Å, 92.5Å, 100Å, 107.5Å, 115Å]. The result of this integration is shown in **Figure S5**, which demonstrates that this results in a model for the hierarchical organization of tau in these NFTs that is consistent with observation.

SAXS scattering includes contributions from other constituents of the lesions as well as that due to voids formed during dehydration of the tissue (17). In many cases, the contribution of other constituents of lesions to the observed scattering is closely similar to that observed in scattering from adjacent tissue. This ‘background’ scattering from tissue provides an estimate of the contribution of other constituents to that observed from lesions.

The correlation between μXRD and μXRF

At high X-ray energy (13keV), the XRF spectrum of heavier elements was collected simultaneously with scanning μXRD. **Figure 1c** exhibits the averaged spectrum from three regions, one identified as tissue, one as low fibrillar tau and one as high fibrillar tau by the integral intensity of β-strands scattering at 1.36 $Å^{-1}$ as detailed in **Figure 1b**. Qualitatively the density of sulfur (S), calcium (Ca), iron (Fe) and zinc (Zn) in the irradiated volume can be obtained by fitting the spectrum with elements determined from **Figure S6**.

Figure S7 includes a superposition of the map of scattering of β-strands (from μXRD) with the maps of Zn and Ca. The map of scattering of β-strands has been normalized and transformed to an RGB image with μXRD as red channel and Zn as green channel, Ca as blue channel. In **Figure S7b** regions corresponding to the deposition of fibrillar tau has a white color, which indicates that the presence of high fibrillar tau with β-strands is associated with deposition of both Zn and Ca. In the Granular layer the strong correspondence of Zn and Ca with low fibrillar tau appears as cyan (light blue) instead of white.

The high resolution XRF analysis.

High resolution XRF image data was obtained by scanning the tissues with a 0.3 × 0.8 μm beam at ID21 ESRF using X-rays with an energy of 4 KeV to emphasize the signal from light elements such as phosphorous, sulfur and calcium. Data was collected on a square grid of 99 x 99 $μm^2$ with step size of 1 $μm^2$. A complete XRF spectrum such as that shown in **Figure S8** is collected at each scan position. The relative abundances of elements at each position are determined by fitting the XRF spectra with multi-Gaussian functions using PyMca software.

Due to differences in experimental setup, the registration of the high-resolution maps of calcium, phosphorous and sulfur in regions R1 to R4 vary slightly from that shown in **Figure S7**. However, the morphologies of cells, particularly those containing fibrillar tau, enabled the alignment of the high-resolution XRF maps with those in R1 to R4 in **Figure S7**. The high resolution XRF maps exhibit more details of the distribution of elements within the cell. Superposition of maps in **Figure S7** demonstrates that calcium and zinc content co-locate with both low-fibrillar and high-fibrillar tau. Region R4 of **Figure 5** and **Figure S7** shows that sulfur co-locates with fibrillar tau. Overlap of the maps of calcium, phosphorous and sulfur demonstrates that calcium and phosphorous are distributed throughout the pyramidal cells, which appears bright white in Region 3 and 4 in **Figure S9**.

bioRxiv preprint doi: https://doi.org/10.1101/2024.11.19.624253; this version posted November 19, 2024. The copyright holder for this preprint (which was not certified by peer review) is the author/funder, who has granted bioRxiv a license to display the preprint in perpetuity. It is made available under aCC-BY 4.0 International license.

Expanded View Figures

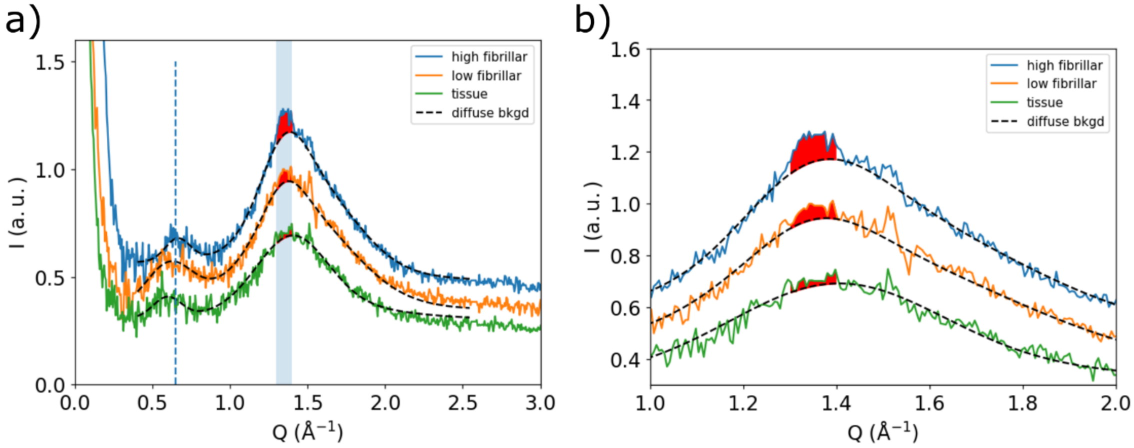

Figure S1. a) Comparison of diffraction from regions of a histological section containing only tissue (green), containing lesions dominated by low-fibrillar tau (orange) and containing lesions composed of fibrillar tau (blue). The smooth background curves (dashed lines) are calculated as a sum of Pseudo-Voigt functions and represent an estimate of the scattering of tissue which is comprised largely of fixed, partially denatured macromolecules. The vertical dashed line at Q = 0.65 $Å^{-1}$ indicates the peak arising from structural features with characteristic dimension of ~ 10 Å. The blue vertical bar highlights the Q range from 1.32 $Å^{-1}$ to 1.4 $Å^{-1}$, which corresponds to structural features with dimensions ~ 4.7 Å. **b)** the deposition of tau enhances the intensity at ~ 1.32 $Å^{-1}$, with progressively greater levels of fibrillation giving rise to a sharper and more intense peak at that position. Scattering from low-fibrillar tau causes the broad, wide-angle peaks to shift from Q = 0.5 $Å^{-1}$ to 0.65 $Å^{-1}$ and Q = 1.42 $Å^{-1}$ to 1.36 $Å^{-1}$. The degree of fibrillation is estimated by subtraction of the diffuse background, resulting in a difference intensity (red) indicative of the degree of tau fibrillation. The broad scattering peak in the wide-angle regime spanning a region from 1.0 < Q < 2.0 $Å^{-1}$ is generated by scattering from disordered, partially denatured and cross-linked macromolecules that make up much of the mass of the fixed tissue.

bioRxiv preprint doi: https://doi.org/10.1101/2024.11.19.624253; this version posted November 19, 2024. The copyright holder for this preprint (which was not certified by peer review) is the author/funder, who has granted bioRxiv a license to display the preprint in perpetuity. It is made available under aCC-BY 4.0 International license.

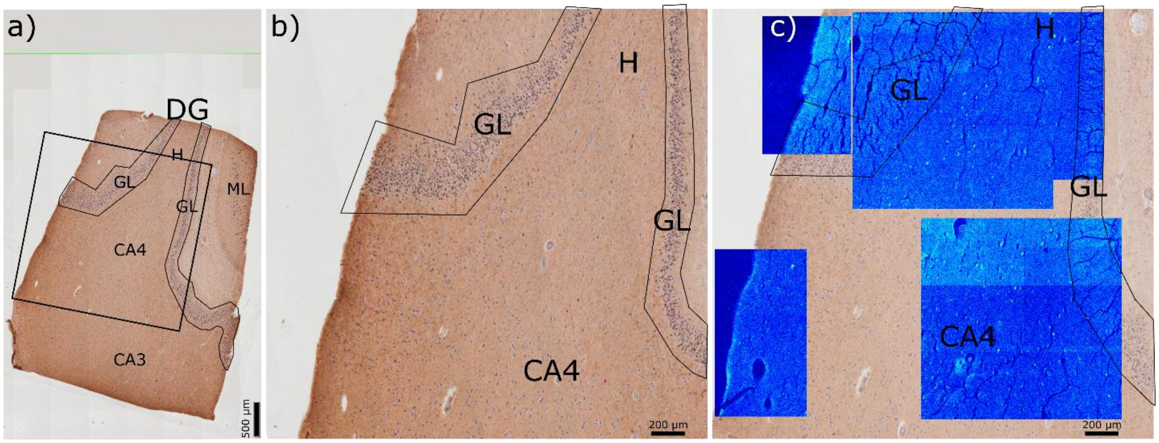

Figure S2. a) An immunostained serial section of tissue from Dentate Gyrus providing a larger anatomical context for the images in **Figure 2** and exhibiting the distribution of tau and its association with tissue morphology. **b)** Enlargement of regions scanned by X-ray microdiffraction. **c)** Maps of the distribution of fibrillar tau determined by the intensity of the 4.7 Å peak (estimated as described in **Figure S1**) superimposed on the image of the immunostained serial section. Lighter blue indicates greater integral intensity. Additional analyses of these ROIs are detailed in **Figure 2**. Abbreviations in the figure: **GL**-Granular Layer, **H**-Hilus, **CA4**- Cornu Ammonis region 4.

bioRxiv preprint doi: https://doi.org/10.1101/2024.11.19.624253; this version posted November 19, 2024. The copyright holder for this preprint (which was not certified by peer review) is the author/funder, who has granted bioRxiv a license to display the preprint in perpetuity. It is made available under aCC-BY 4.0 International license.

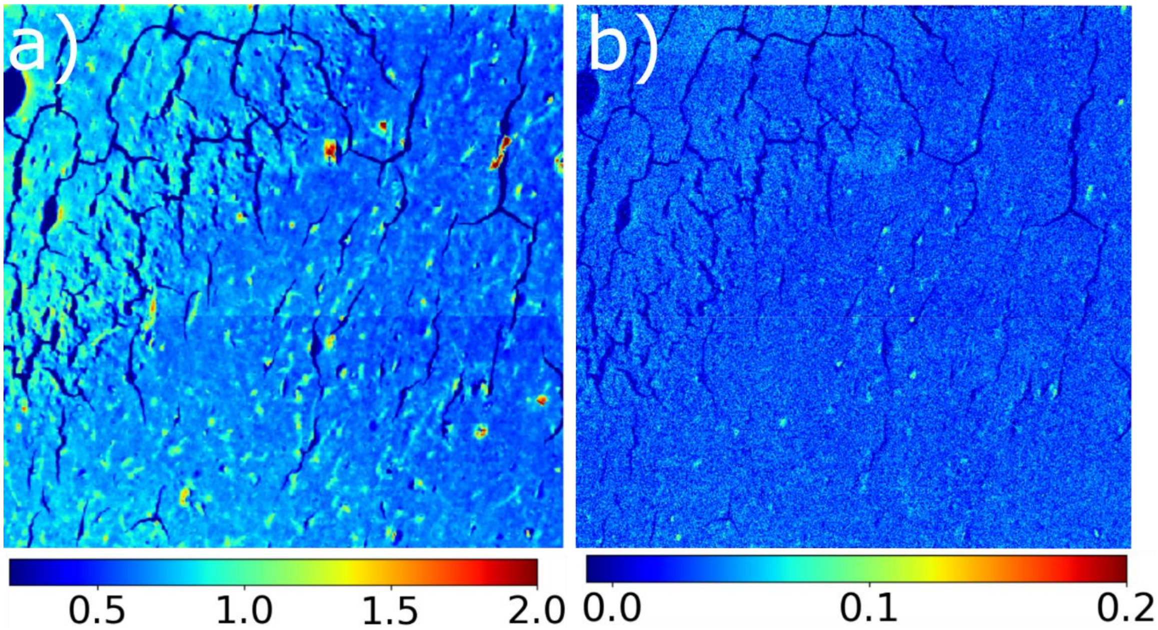

Figure S3. **a)** The map of the integral intensity of scattering from the Q range from 1.32 Å^{-1} to 1.4 Å^{-1} allows visualization of the distribution of molecular mass for the proteinaceous tissue in the Dentate Gyrus. The distribution includes many peaks that correspond to tau-containing lesions but does not distinguish between lesions with low or high levels of fibrillar tau. **b)** the distribution of fibrillar tau in the tissue as estimated by subtraction of diffuse tissue scattering as detailed in **Figure S1**. Lighter blue indicates greater concentration of fibrillar tau. Fibrillar tau is concentrated in punctate features prominent in (b) and is largely absent from the granular layer that occupies the upper right in these images.

bioRxiv preprint doi: https://doi.org/10.1101/2024.11.19.624253; this version posted November 19, 2024. The copyright holder for this preprint (which was not certified by peer review) is the author/funder, who has granted bioRxiv a license to display the preprint in perpetuity. It is made available under aCC-BY 4.0 International license.

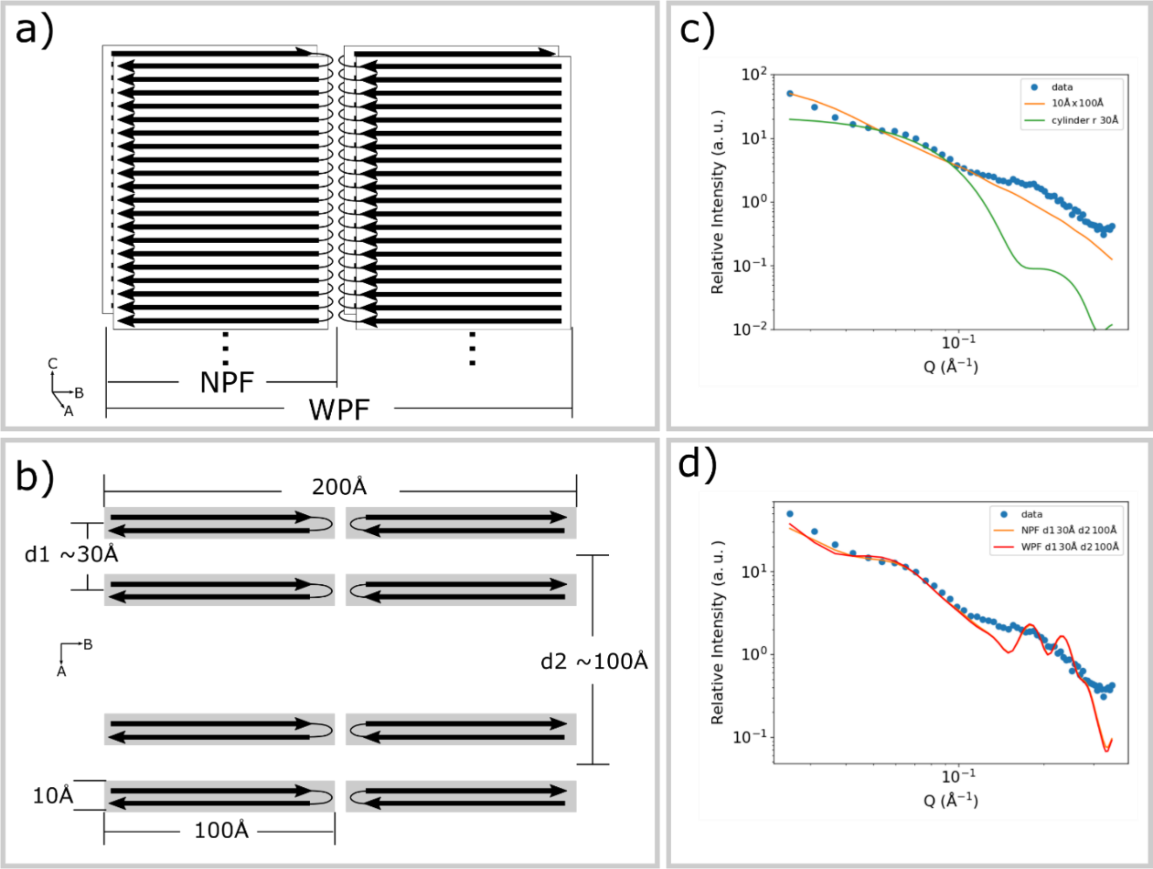

Figure S4. Constructing a hierarchical model of fibrillar Tau to fit the observed SAXS data. a) The fibril model is built on the basis of the structure of narrow fibrils (NF) determined by cryoEM. The wide fibrils are comprised of two narrow fibrils related to one another by a two-fold axis parallel to the fiber axis. b) Models constructed as hierarchical aggregates of narrow fibrils, including wide fibrils, exhibit organization on at least two length scales. The first is formed from the intra-fibril distance, d1, of ~ 30 Å and the second from inter-fibril distances, d2, of ~ 100 Å. c) SAXS calculated from the Narrow Fibril model (red) exhibits a slope comparable to that observed, but lacks the distinctive shoulders. A cylinder model (green curve) proposed earlier, (18), reproduces some of the SAXS features but does not predict the overall decrease of intensity as a function of Q. d) The calculated SAXS profile for these choices of d1 and d2 fits the first intensity shoulder well, but not the second.

bioRxiv preprint doi: https://doi.org/10.1101/2024.11.19.624253; this version posted November 19, 2024. The copyright holder for this preprint (which was not certified by peer review) is the author/funder, who has granted bioRxiv a license to display the preprint in perpetuity. It is made available under aCC-BY 4.0 International license.

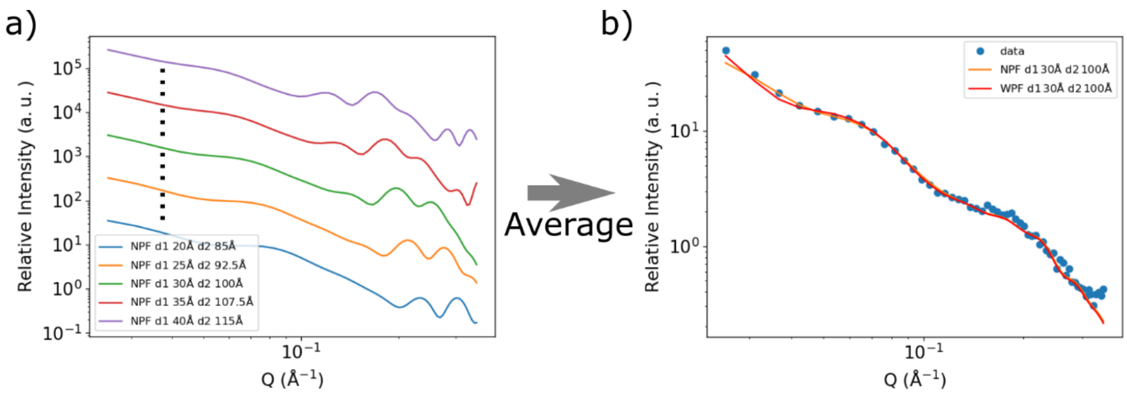

Figure S5. Adding polydispersity to the multi-scale hierarchical model for Tau filaments. a) For any given choice of d1 and d2, the calculated SAXS profiles include features not consistent with experiment. b) An ensemble of fibrillar aggregates constructed with d1 varying from 20 – 40 Å and d2 from 85 to 115 Å with equal probability, results in a SAXS pattern consistent with that observed *in situ* strongly suggesting that *in situ* the fibrillar aggregates are organized in a hierarchical structure that exhibits limited variation of fibril-fibril distances.

bioRxiv preprint doi: https://doi.org/10.1101/2024.11.19.624253; this version posted November 19, 2024. The copyright holder for this preprint (which was not certified by peer review) is the author/funder, who has granted bioRxiv a license to display the preprint in perpetuity. It is made available under aCC-BY 4.0 International license.

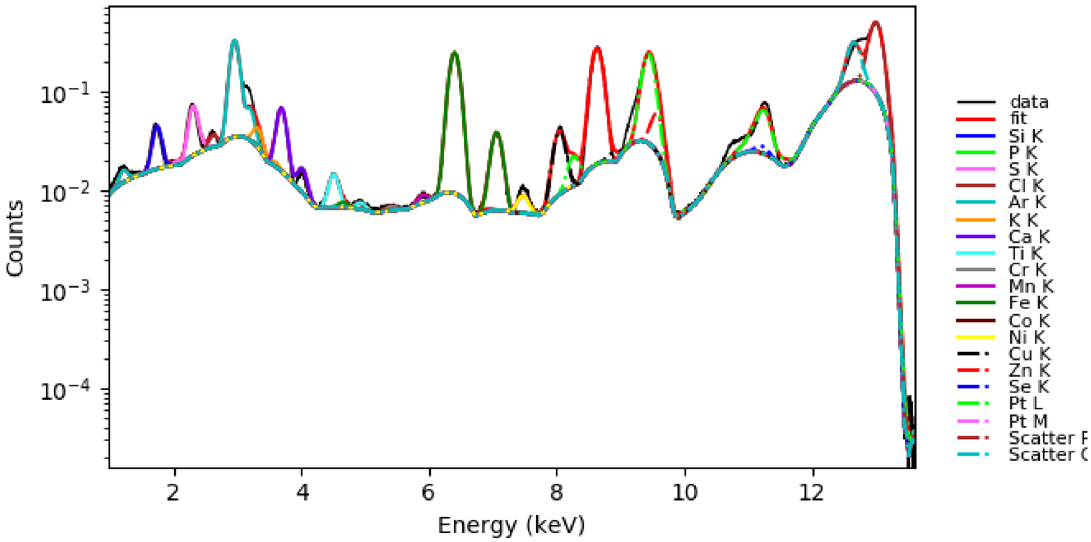

Figure S6. The averaged XRF spectrum from ID13 µXRF image map, with peak heights of individual elemental peaks estimated using PyMca

bioRxiv preprint doi: https://doi.org/10.1101/2024.11.19.624253; this version posted November 19, 2024. The copyright holder for this preprint (which was not certified by peer review) is the author/funder, who has granted bioRxiv a license to display the preprint in perpetuity. It is made available under aCC-BY 4.0 International license.

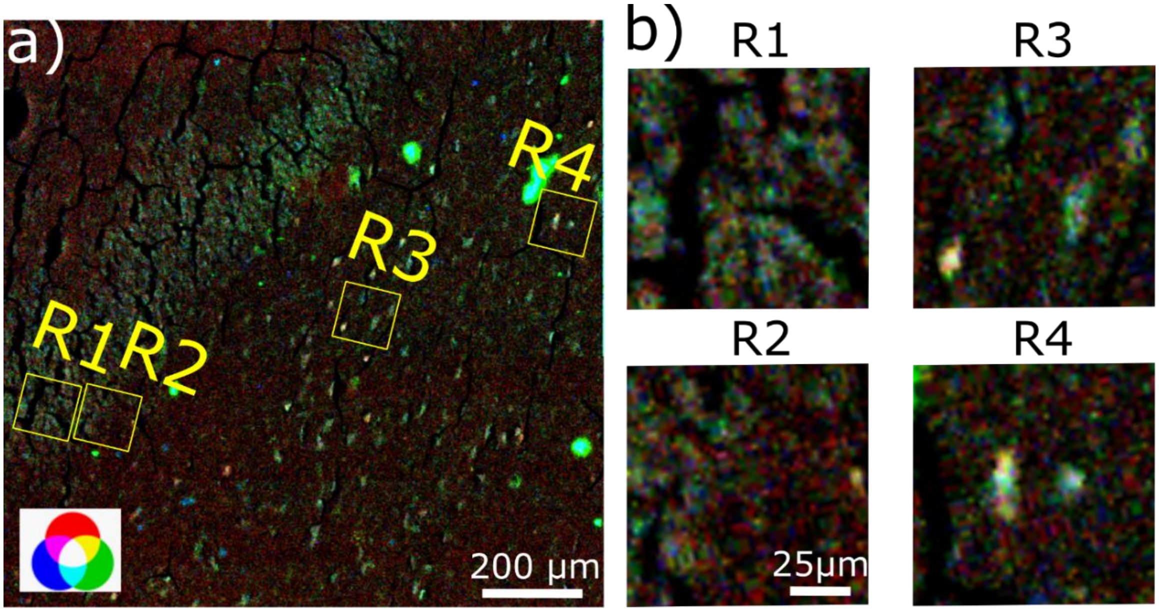

Figure S7. Superposition of the distribution of total tau estimated from the scattering at 4.7 Å with the distribution of Zn and Ca from XRF. (a) includes the entire ROI exhibited in **Figure 2**. (b) includes enlargements of four regions within that ROI. R1 and R2 in the granular layer exhibit strong co-deposition of Zn and Ca with low-fibrillar tau as visualized by the superposition of green and blue leading to cyan or light blue color in the enlargements on the right. In R3 and R4, in the hilus, places where fibrillar tau co-localizes with Zn and Ca appear as white. The label in **a)** shows the mixture of RGB colors.

bioRxiv preprint doi: https://doi.org/10.1101/2024.11.19.624253; this version posted November 19, 2024. The copyright holder for this preprint (which was not certified by peer review) is the author/funder, who has granted bioRxiv a license to display the preprint in perpetuity. It is made available under aCC-BY 4.0 International license.

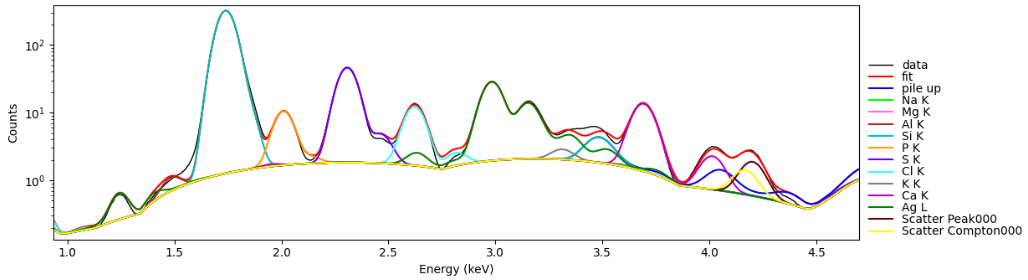

Figure S8. The XRF spectrum from ID21 µXRF image map, fitted using PyMca

bioRxiv preprint doi: https://doi.org/10.1101/2024.11.19.624253; this version posted November 19, 2024. The copyright holder for this preprint (which was not certified by peer review) is the author/funder, who has granted bioRxiv a license to display the preprint in perpetuity. It is made available under aCC-BY 4.0 International license.

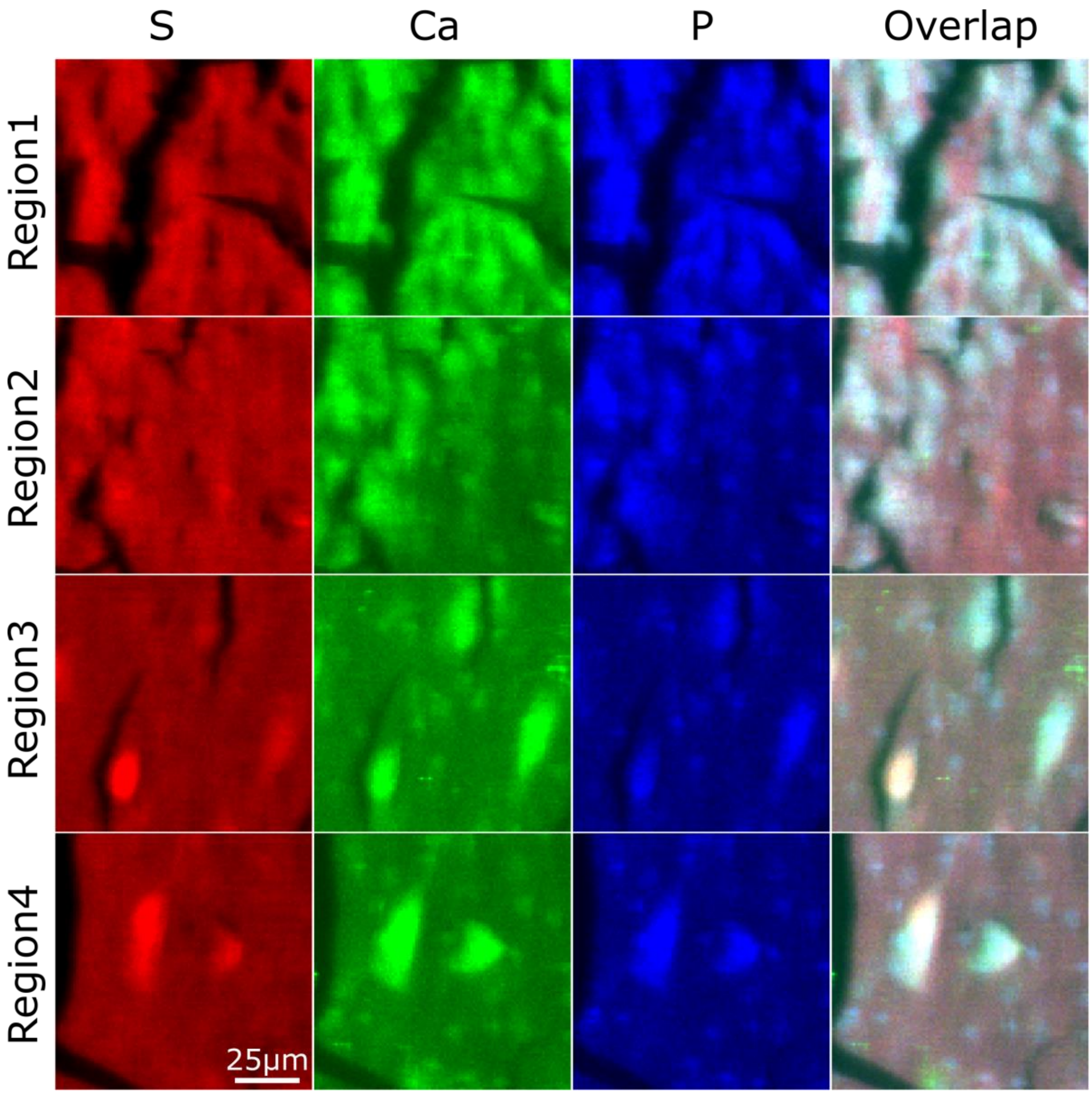

Figure S9. Comparison of the distribution of elements from high-resolution XRF data. Here, the maps in **Figure 5** of main text are transformed to RGB format. The map of sulfur is transformed to the red channel, calcium to the green channel, and phosphorous to the blue channel. Both calcium and phosphorous exhibit deposition in all tau-containing lesions. Sulfur demonstrates strong correlation with calcium and phosphorous only in Regions 3 and 4, indicating a strong co-localization with fibrillar tau.

bioRxiv preprint doi: https://doi.org/10.1101/2024.11.19.624253; this version posted November 19, 2024. The copyright holder for this preprint (which was not certified by peer review) is the author/funder, who has granted bioRxiv a license to display the preprint in perpetuity. It is made available under aCC-BY 4.0 International license.

Appendix References

1. Falcon, B., Zhang, W., Murzin, A. G., Murshudov, G., Garringer, H., Vidal, R., . . . Goedert, M. (2018). Structures of filaments from Pick's disease reveal a novel tau protein fold. *Nature, 561*(7721), 137-140.
2. Nepal, P., Bashit, A. A., & Makowski, L. (2024). Small-angle X-ray microdiffraction from fixed human brain tissue exhibits power-law behavior that provides insights into the structural organization of neuropathological lesions. *Biophysical Journal, 123*(3), 135a.
3. Schweers, O., Schonbrunn-Hanebeck, E., Marx, A., & Mandelkow, E. (1994). Structural Studies of Tau Protein and Alzheimer Paired Helical Filaments Show No Evidence for beta-Structure. *The Jounral of Biological Chemistry, 269*(39), 24290-24297.
